# On the Influence of Slight Modifications of Culture Media on the Growth of Bacteria as Illustrated by the Glanders Bacillus

**Published:** 1890-03

**Authors:** Theobald Smith

**Affiliations:** The Bureau of Animal Industry, Department of Agriculture, Washington, D. C.


### ON THE INFLUENCE OF SLIGHT MODIFICATIONS OF CULTURE MEDIA ON THE GROWTH OF BACTERIA AS ILLUSTRATED BY THE GLANDERS BACILLUS.

By THEOBALD SMITH, M.D.,
*Of the Bureau of Animal Industry, Department of Agriculture, Washington, D. C.*

WHILE carrying on some experiments to determine the value of the inoculation of guinea-pigs with nasal mucus in the diagnosis of equine glanders[1] I was impressed with the feebleness of the growth of glanders bacilli in ordinary culture media, such as agar and bouillon-peptone. The use of glycerine as a valuable addition to culture media for glanders bacilli was introduced by Kranzfeld.[2] The richness of the growth on agar to which 5 per

[1] Annual report of the Dept. of Agriculture for 1888, p. 206–219.
[2] Centralbl. f. Bacteriologie, 1887, II., 273.

cent. glycerine has been added is very marked when compared with the growth in the same media not containing glycerine. In the modification of the culture media I went a step farther and used them not neutralized, *i. e.*, with a *slightly acid reaction*. The effect of this change was to increase to a considerable degree the richness of the growth as observed on alkaline media containing glycerine. The idea of using slightly acid media was suggested to me by the very rich growth which glanders bacilli indulge in on potato, which has usually a well-marked acid reaction. I was moreover puzzled by the fact that while the potato culture has a decided color suggesting the red of cuprous oxide (Löffler), the growth on alkaline agar with or without glycerine has only a gray or whitish color.

Anticipating somewhat the descriptions to follow, I might state here that in comparing parallel cultures from at least half a dozen guinea-pigs inoculated with the nasal mucus of as many different horses, I noticed more or less variation in the appearance of the cultures. These variations were transmitted from culture to culture, and hence cannot well be regarded as due to slight variations in the culture media employed. In presenting a brief description of the culture appearances in acid glycerine media I shall refer to these variations in detail.

When the inclined surface of acid glycerine agar[1] is inoculated from the pus of the testicle or lymphatic glands of a glandered guinea-pig, the isolated colonies which appear within twenty-four to thirty-six hours in the thermostat, are circular, grayish, translucent, from 1½ to 2 mm. in diameter. When successive inoculations are made, the growth of the later generations becomes richer and forms either a shining, smooth membrane on the surface of the agar or a finely wrinkled one having the appearance of ground glass. This wrinkling is due to the very abundant growth, which is really as rapid as that of any germ with which I am acquainted. The growth on alkaline glycerine agar is generally but half as dense. The growth in *acid bouillon* with peptone, salt and 5 per cent. glycerine is even more striking. In the first generation it is apt to be feebler than in the succeeding ones. During the week following the inoculation the growth is slight. It appears in the

[1] The acidity of the media employed is that of fresh beef infusion plus the acid which may be contained in 1 per cent. peptone, ½ per cent. agar and ½ per cent. sodium chloride. The glycerine used was the best obtainable. I am unable to state whether similar success would attend the use of meat extracts.

form of isolated points on the surface with slight clouding of the liquid. Suddenly, after the tube has been quiet in the thermostat for a few days, a membrane is observed, often rising on the sides of the tube above the liquid and at first simulating the membrane of *bacillus subtilis*. The membrane grows more fleshy, festoons and shreds hang down from it into the now quite turbid liquid. When the tube is shaken the entire membrane masses into a viscid ball which gradually settles to the bottom. Within a few days another complete membrane may have formed. The deposit in the bottom of the tube accumulates until more abundant than that formed by most saprophytes. This remarkably luxuriant growth is the result of the combined effect of a slightly acid medium and glycerine. Thus it is less abundant when the liquid is alkaline. It is quite feeble in acid bouillon peptone without glycerine, and in the same fluid made alkaline no growth may appear at all.

In connection with the above observations, the color of the growth aroused attention. The acid agar culture usually assumed a pale straw color, while in the bouillon the membrane became decidedly orange. This pigment did not appear in alkaline media nor in acid cultures from all sources. Those bacilli which showed the orange tint retained it in all subsequent cultures. In cultures from one source a tendency to form a pigment was never manifested. The color thus varied from a translucent grayish to a cream and orange tint. This variation in the depth of the color I have observed on potatoes, and it is probably inherent in the glanders bacilli. The formation of the pigment seems to be connected with the destruction of the bacilli in the culture media. In agar cultures in which the upper portion of the inclined surface has become dried up the colonies on this surface have a brick-red color, though the colonies below are grayish. Whatever may be the origin of the pigment, it is an element of varying intensity and this must not be lost sight of in the diagnosis of cultures. In general it may be said that if the bacillus forms a well-marked pigment it will appear in acid glycerine media as it does on potato.

An interesting fact brought out by the liquid cultures is the almost exclusive surface growth of the bacilli, thus betraying their demand for oxygen. The viscid character of the growth pointed out by Löffler in agar cultures is very manifest in liquid media. It is often difficult to break or tear off with the platinum loop a portion of the membranous growth.

As an illustration of the specific character of the bacillus possessing the characters described, I quote the following experiment :—

March 14th, guinea-pig inoculated subcutaneously from nasal discharge of glandered horse, killed April 23d. The left testicle converted into a large unbroken abscess. Spleen enlarged ; free from nodules. A potato and an alkaline glycerine agar culture from the pus of the testicle develop ; the latter into a thick *whitish* viscid layer ; the former into a *brownish-red* mass. May 3d, a number of tubes containing acid glycerine bouillon inoculated from the agar culture. May 16th, a complete, wrinkled, cup-shaped membrane of a pale *orange* color on the liquid. May 24th, the membrane shaken down a few days ago has been replaced by another. June 4th, from the viscid orange deposit in one liquid culture two guinea-pigs are inoculated subcutaneously. One recovers ; the other is killed June 18th. Large subcutaneous abscess near place of inoculation. Left testicle contains a large abscess. Left forefoot and right hindfoot swollen ; the latter ulcerated. Cultures from spleen in part sterile. Those from the pus of testicle on potato characteristic ; in the acid glycerine media the color of the rapid, abundant growth very pronounced. In an Esmarch tube of acid glycerine agar from the pus three or four surface colonies appear, 1-2 mm. in diameter, consisting of a wrinkled, faintly yellow surface growth.

In the study of glanders bacilli we are forced to depend more or less on the macroscopic appearances of cultures, owing to the great variety of puzzling involution forms which appear in cultures and which make the microscopic examination, taken alone, of little value. The use of acid media containing glycerine will, I think, materially assist us in this direction. It also indicates how it may be necessary to proceed in endeavoring to find suitable substrata for the multiplication of those still refractory organisms of tuberculosis, leprosy and the like. The generally accepted view that bacteria prefer alkaline media must be modified into the statement that some bacteria prefer alkaline, others slightly acid media.

The foregoing observations on glanders bacilli, briefly summed up, have shown that slightly acid media are much better than alkaline ; that they bring out the pigment not observed in alkaline media. They have demonstrated the abundant growth in liquids with its tendency to remain restricted to the uppermost strata, its cohesive nature and the variation in the amount of pigment produced. Lastly, the cultures remain alive much longer than on other media, a matter of some importance in bacteriological laboratories.

